# Characteristics of Atopic Bronchial Asthma in Seniors over 80 Years of Age

**DOI:** 10.1155/2013/689782

**Published:** 2013-07-31

**Authors:** Andrzej Bożek, Marek Filipowski, Andreas Fischer, Jerzy Jarzab

**Affiliations:** ^1^Clinical Department of Internal Medicine, Dermatology and Allergology, Medical University of Silesia, M. Sklodowskiej-Curie 10, 41-800 Zabrze, Poland; ^2^Clinical Department of Chest Surgery, Medical University of Silesia, 41-880 Zabrze, Poland; ^3^Genetic Laboratory MTD, 80-331 Munchen, Germany

## Abstract

*Background*. Asthma in the elderly is an important public health problem. The aim of this study was to assess the prevalence and characteristics of asthma in seniors. *Materials and Methods*. The study involved 105 people of at least 80 years of age (mean age of 84.1 ± 3.9 years) selected from a group of 1860 individuals. Spirometry, the methacholine test, allergy diagnosis, a measurement of exhaled nitric oxide, and administration of the asthma quality of life questionnaire (AQLQ) were performed. *Results*. The average morbidity of asthma in the study population of elderly people (at least 80 years of age) was 5.6% (105 people) of the confidence interval (95% CI: 5.1–6.0). In the study group, 34% of the elderly asthmatics had uncontrolled asthma, 47% had partly controlled asthma, and only 24% had fully controlled asthma. Allergy to house dust mites was predominant. The average total score on the AQLQ was 4.12 ± 0.72 (arithmetic mean ± standard deviation) for the seniors, which was significantly lower than the score for the young. *Conclusion*. The pathogenesis, natural history, and value of the basic diagnostic methods of asthma in the elderly are similar to those observed in younger age groups.

## 1. Background

Bronchial asthma is an increasing problem in all age groups. However, there is still not enough information about the natural course and clinical features of asthma in seniors. Additionally, a clear diagnosis of asthma in patients over 80 years of age is difficult [1, 2]. Similarities between COPD and asthma, overlap syndrome and the presence of other diseases make it difficult to diagnose asthma in elderly patients. Many older patients are still underdiagnosed [1–4]. This phenomenon results in the undertreatment of asthma and leads to a deterioration in quality of life [4]. This group of patients is frequently incorrectly diagnosed with COPD, and seniors are unwilling to undergo diagnostic tests for allergy [3, 5].

The aim of the study was to assess the differences between the clinical features of asthma in patients over 80 years old and youth. This study could successfully draw the necessary attention to the special needs of these patients.

## 2. Materials and Methods

The study group enrolled 105 patients who were at least 80 years old and had a preliminary diagnosis of asthma. The recruitment of respondents was conducted in eight centers representative of the Polish population. Stratification sampling was performed from 1860 patients in the key age group of over 80 years of age. The control group consisted of young patients diagnosed with asthma and selected from GP clinics in the same areas of residence as the group of elderly patients. Stratification sampling was also performed, with a key age group of 18–30 years of age. Asthma was diagnosed on the basis of the ECRHS II questionnaire [6], a physical examination, and spirometry with a positive result for the reversibility test (Lungtest 1000, MES, Krakow, Poland) or with a positive result for the methacholine test. For a positive test result, an increase in the forced expiratory volume in one second (FEV1) of ≥12% or an increase of 200 mL was necessary, in accordance with the criteria of the ATS and ERS [7]. Before testing, the patients did not use a short-acting beta2 agonist for 8 hours, ipratropium and theophylline derivatives for 24 hours, a long-acting beta2 agonist for 48 hours, or tiotropium for 72 hours. Current smokers refrained from smoking for 1 hour before testing. Testing was delayed for 4 weeks in the case of respiratory tract infections. The methacholine test was performed based on the standards of the Polish Society of Allergology [8]. A positive result for the test corresponded to a decline in the FEV1 of 20% and a PC20 of <8 mg/mL. The subjects completed the asthma control test (ACT) [9]. The asthma severity and control of the disease were determined on the basis of an interview and spirometry test and assessed according criteria of Global Initiative for Asthma (GINA) [10]. Patients with negative reversibility test and with clinical manifestations of COPD or asthma/COPD overlap syndrome were excluded from the study. All subjects signed a consent form, agreeing to participate in the study.

## 3. Allergy Testing

### 3.1. Concentration of IgE

Measurement of the concentration of total IgE and allergen-specific IgE (sIgE) was performed using the Pharmacia CAP System FEIA (Pharmacia AB, Uppsala, Sweden), and the values are shown in kU/l [11]. Research was performed for the following specific IgE allergens: *Dermatophagoides pteronyssinus*, *Dermatophagoides farinae*, *Aspergillus fumigatus*, *Alternaria tennis*, *Cladosporium herbarum*, dog dander, cat dander, grass mix, birch, alder, hazel, and mugwort. IgE values above 0.35 kU/l were considered to be a positive result [11].

### 3.2. Skin Prick Tests (SPTs)

In the study, SPTs were obtained from Allergopharma (Reinbeck, Germany), corresponding to a set of allergen-specific IgEs. The tests were performed according to the guidelines [12].

### 3.3. Exhaled Nitric Oxide

The level of fractional exhaled nitric oxide (FENO) was assessed in the senior group of patients and the young asthmatic control group. The determination of FENO was performed using a manual analyzer, the MINO Airway Inflammation Niox Monitor (Aerocrine AB, Solna, Sweden), measuring the exhaled fraction of nitric oxide by an electrochemical method [13]. The patients performed three repeated attempts to exhale into a mouthpiece device while maintaining a constant positive pressure between 12 and 18 cm water head to maintain a flow of air of 50 ± 5 mL/s, in accordance with the recommendations of the American Thoracic Society [14]. The patients did not smoke cigarettes for 20 minutes before the test, did not eat foods rich in nitrogen for one day before the test, and did not take inhaled drugs (beta2 agonists and inhaled steroids) for 6 hours before the test. The study excluded patients with symptoms of respiratory infection. FaENO values were reported in parts per billion (ppb). The value ranged from 5 to 300 ppb. For this analysis, the mean of three reproducible results were used. 

A patient quality-of-life assessment was performed using the asthma quality of life questionnaire (AQLQ) developed by Juniper, whose application was approved. The questionnaire included questions about the group: the environmental exposure of the patients, the reduction in physical activity by the disease, the patient's emotional evaluation in the context of the disease, and the symptoms of the disease itself. For each of the 32 questions, the patient responded on a scale of 1–9 points, with the lowest to the highest value representing the worst to the best assessment. Details of the questionnaire can be found in the literature [15].

### 3.4. Statistical Analysis

For comparison, data meeting the criterion of a normal distribution, such as the age of the participants, the duration of the disease, and the results of the spirometry tests, were analyzed using a Student's *t*-test for independent samples. To assess the correlation of the results of the skin tests and IgE measurements, Spearman's test was used. In addition, the logarithms of the IgE values met the criterion for a normal distribution, so Pearson's correlation test was used. Odds ratios were calculated to assess the likelihood of asthma in the presence of a particular trait in the subjects. Multiple linear regression analysis was used for the dependent variable, which was the duration of asthma, and for independent variables, such as smoking, the IgE value, asthma control, the ACT score, the severity of asthma, and the presence of allergic rhinitis. The statistical analyses used a Bonferroni correction. A *P* value of <0.05 was considered significant.

The study was approved by the Bioethical Committee of the University Medical School of Silesia in Katowice, Poland.

## 4. Results

Based on data from 1860 screened individuals, the prevalence of asthma associated with age and sex in the study population of elderly people (at least 80 years of age) was 5.6% (105 people) of the confidence interval (95% CI: 5.1–6.0). There were 63 women and 42 men with a mean age of 84.1 ± 3.9 years (range of 80 to 97 years). The control group included 102 young patients with a mean age of 22.1 ± 5.2 years (range of 18 to 32 years), including 45 women and 67 men.

The demographic data for the patients and the degree of the control of asthma are shown in Table 1.

The mean spirometric values were as follows: mean FEV1 in elderly asthmatics: 1.21 ± 0.45 L (mean 68.4% of the predicted value); mean FVC in elderly asthmatics: 1.73 ± 0.43 L (77.4% of predicted value); mean FEV1 in young asthmatics: 1.87 ± 0.49 L (73.1% of predicted value); and mean FVC in young asthmatics: 2.31 ± 0.69 L (83.2% of predicted value). There was a significant difference between the elderly and youth in absolute value (*P* < 0.05). However, there was no statistical difference between percentage of normal range according to age which was obtained by elderly and young. An allergy to the house dust mite *D. pteronyssinus* and/or *D. farinae* (positive skin tests and corresponding IgE) was predominant in approximately 47% and 45% of the young and old patients, respectively. This followed by a grass pollens (27% in elderly versus 24% in young), tree pollens (29% versus 28%), cockroach (21% versus 19%), molds (15% versus 9%), and animal dander (10% versus 9%). The results of the tests, including for specific IgE, were comparable between the two groups, except for a significant predominance of allergy to molds in the elderly (*P* < 0.05). 

The analysis of the impact of various factors on the presence of asthma in studied subjects were performed and showed in Table 2.

The total serum IgE was 189.4 ± 38.7 kU/L in elderly and 201.7 ± 78.9 kU/L in control group. There was no statistical difference.

In all patients, the initial diagnosis of seniors was performed with an asthma reversibility test, which was positive in 86 (78%) patients (increase in the FEV1 of ≥12% or an increase of 200 mL). In total, 11 (10.5%) patients had negative result of reversibility test. In 8 (7%) patients, the test could not be performed because of a lack of cooperation. Patients in the last two subgroups (11 plus 8 people) underwent assessment with a test for methacholine bronchial hyperreactivity, which indicated that 14 (13.3%) patients met the criterion for the diagnosis of asthma (FEV1 decline of 20%). Due to a low FEV1 value (<70% of normal and less than 1 l), 17 patients withdrew from the study. Finally, 5 (4.8%) patients were classified into groups with COPD or asthma/COPD overlap syndrome (characteristic clinical manifestation, changes in X-ray, negative reversibility test). They were excluded from the study. The evaluation of the measurement of nitric oxide is shown in Table 3.

The average total score on the AQLQ was 4.12 ± 0.72 (arithmetic mean ± standard deviation) for seniors, which was significantly lower than the score for the youth: 5.12  ±  0.48 (Student's *t*-test, *P* < 0.05). A similar trend was observed for each of the analyzed domains: environmental exposure: 4.13 ± 0.53 in seniors versus 5.16 ± 0.33 in young (*P* < 0.05); emotional function: 4.32 ± 0.61 versus 4.90 ± 0.34 (*P* < 0.05); and activity: 4.01 ± 0.55 versus 5.4 ± 0.63 (*P* < 0.05). The quality of life was significantly correlated with better asthma control according to the ACT (Spearman's rank correlation test, *R* = 0.87, *P* < 0.05). AQLQ scores did not correlate with spirometric values (FEV1).

## 5. Discussion

The obtained results showed lack of significant differences between clinical features of asthma in seniors and young. The severity of the disease, the result of ACT, and the degree of asthma control were similar to young patients. This phenomenon is interesting and contradicts the observations between asthmatic patients aged 50–70 years compared with the young as it was confirmed in previous studies [16, 17] where more severe and frequent uncontrolled asthma in older patients was noticed. This can be explained by the overall good health condition of the respondents, which allowed them to survive more than 80 years of age. Lifestyle and genetic factors may play a fundamental role here. The analyzed group of patients, with asthma and over 80 years of age, is only one of such study. The other studies did not analyse homogenous populations, and majority of patients were in age range 50–80 [16–20]. The prevalence of asthma observed in the study did not differ significantly from the data available in the literature [18–20]. Among the analyzed patients with asthma, the percentage of those who were women was 56%. This finding is a consequence of the predominance of women in the general population of seniors. The distribution of asthma presented in the literature, characterized by a long duration of the disease or a late-onset form, was also reflected in this study [17]. Nearly 18% of patients experienced asthma symptoms at 65 years of age. Despite reports [16, 21, 22] of their differences in relation to long-duration asthma, such as milder, more asymptomatic days; less hospitalization; and improved spirometric parameters, these features were not observed in the current study. Confirmed great influence atopy on the development of asthma is well documented also in other studies, although in a slightly younger age groups [22–24]. This is another evidence of the nature of asthma, regardless of age. Allergic profile with domination of house dust mite allergy was comparable with results of other authors [23, 24]. On the other hand, many studies have shown a low reactivity of skin to testing and decrease in IgE-dependent activity in the elderly. However, it did not negate the importance of diagnostic IgE mediated reaction in this group [16, 17]. The comparable value of total IgE in elderly and young confirmed the consistent prevalence of allergy, regardless of age. It was opposite to other observations [16, 17]. Additionally, the results showed that smoking is not such a problem as it is commonly thought in patients with asthma. We also did not observe any coincidences between smoking and course of asthma and value of IgE. It was faced with conflicting evidences [19–21]. This may be due to very old age.

The performed pulmonary function studies highlighted the value of spirometry in the diagnosis and monitoring of asthma in older patients. There was no significant difference in reversibility tests and obtained percentage of normal ranges of of FEV1 and FVC. However, there are differences between absolute values of FEv1 and FVC as it was in previous observations [17, 18]. The lower absolute values observed in older asthmatics than in young asthmatics are proof of the aging process of the respiratory system, with an additional loss of capacity due to prolonged illness. This finding was indicated by previous reports [16–18]. Few studies have raised the issue of performing spirometry in the elderly. Chotirmall indicated the importance of this research to the diagnosis and assessment of asthma in the elderly but also emphasized possible limitations, such as poor cooperation due to cognitive impairment, poor psychomotor coordination, problems with the foundation of the mouthpiece, impaired perception, a poor response to beta2 agonists, and a lack of capacity to perform forced expiration [17]. Pezzoli et al. emphasized the effectiveness of the implementation of spirometry in the elderly [25]. The use of nitric oxide measurements to assess bronchial asthma in the elderly, based on the results, appears to be useful. The higher obtained FENO in the elderly patients may be an evidence of much more advanced inflammation in the airways. This phenomenon may be a consequence of long-term asthma, which is often under-treated, and of systemic subinflammation in the elderly. There was no comparable data in this age range in other studies.

The worse quality of life in seniors with confirmed asthma than in the young may be an evidence for the poorer diagnosis and treatment of and interest in this group of patients. It is emphasizes earlier [17, 19].

## 6. Conclusion

Asthma in the elderly is an important public health problem. This condition's pathogenesis and natural history and the value of basic diagnostic methods are similar to those observed in younger age groups. There is a need to pay more attention to the disease in patients over 80 years old, which would prevent the underdiagnosis and undertreatment of asthma. 

## Figures and Tables

**Table 1:** Clinical characteristic of asthma: comparison between seniors and youth.

Characteristics	Seniors *n* = 105	Youth *n* = 102	*P *
Mean age	84.1 ± 3.9	22.1 ± 5.2	<0.05
Women (%)	63 (60)	45 (44)	<0.05
Smokers (%)	7 (6.7)	9 (8.2)	NS
Former smokers (%)	13 (12,4)	6 (5.9)	NS
Duration of asthma	29.2 ± 5.1	2.4 ± 1.6	<0.05
Late onset of asthma (after 65 years; %)	68 (17.6)	—	—
Mean result of ACT	13.9 ± 4.8	15.7 ± 3.7	NS
Severe asthma (%)	32 (8.4)	23 (5.7)	NS
Moderate asthma (%)	38 (36)	29 (28.8)	NS
Mild asthma (%)	43 (40.6)	43 (41.8)	NS
Fully controlled asthma	24 (22.8)	48 (46.2)	<0.05
Partly controlled asthma	47 (44.8)	49 (48)	NS
Uncontrolled asthma	34 (32.2)	6 (5.8)	<0.05
Allergic rhinitis	59 (56.3)	63 (61.7)	NS

NS: not significant.

**Table 2:** The odds ratio for asthma in elderly patients with analysis various factors.

Factors	Odds ratio (95% CI)
History of atopy at a younger age	1.54 (1.36–1.78)
A positive result for at least one skin test	2.01 (1.86–2.11)
A positive allergen-specific IgE test	2.08 (1.98–2.56)
Smokers	1.88 (1.75–1.91)
Presence of asthma in at least one family member	1.47 (1.26–1.62)
Men	1.49 (1.23–1.98)

**Table 3:** Comparison of exhaled nitric oxide in young and old patients with asthma.

Subjects	FENO (ppb), seniors (mean ± SD)	FENO (ppb), youth (mean ± SD)	*P* ^∧^
All with asthma	41.2 ± 15.04	32.74 ± 12.62	<0.05
Smokers with asthma	46.09 ± 25.33	39.09 ± 15.66	<0.05
Nonsmokers with asthma	38.03 ± 13.2	27.8 ± 11.09	<0.05

^*∧*^Student's *t*-test for unrelated samples with Bonferroni correction.
